# Evidence of Interdomain Ammonium Cross-Feeding From Methylamine- and Glycine Betaine-Degrading *Rhodobacteraceae* to Diatoms as a Widespread Interaction in the Marine Phycosphere

**DOI:** 10.3389/fmicb.2020.533894

**Published:** 2020-10-06

**Authors:** Karsten Zecher, Kristiane Rebecca Hayes, Bodo Philipp

**Affiliations:** Institute for Molecular Microbiology and Biotechnology, University of Münster, Münster, Germany

**Keywords:** methylamines, *Rhodobacteraceae*, diatoms, cross-feeding interaction, phycosphere, glycine betaine

## Abstract

Dissolved organic nitrogen (DON) compounds such as methylamines (MAs) and glycine betaine (GBT) occur at detectable concentrations in marine habitats and are also produced and released by microalgae. For many marine bacteria, these DON compounds can serve as carbon, energy, and nitrogen sources, but microalgae usually cannot metabolize them. Interestingly though, it was previously shown that *Donghicola* sp. strain KarMa—a member of the marine *Rhodobacteraceae*—can cross-feed ammonium such that the ammonium it produces upon degrading monomethylamine (MMA) then serves as nitrogen source for the diatom *Phaeodactylum tricornutum*; thus, these organisms form a mutual metabolic interaction under photoautotrophic conditions. In the present study, we investigated whether this interaction plays a broader role in bacteria–diatom interactions in general. Results showed that cross-feeding between strain KarMa and *P. tricornutum* was also possible with di- and trimethylamine as well as with GBT. Further, cross-feeding of strain KarMa was also observed in cocultures with the diatoms *Amphora coffeaeformis* and *Thalassiosira pseudonana* with MMA as the sole nitrogen source. Regarding cross-feeding involving other *Rhodobacteraceae* strains, the *in silico* analysis of MA and GBT degradation pathways indicated that algae-associated *Rhodobacteraceae*-type strains likely interact with *P. tricornutum* in a similar manner as the strain KarMa does. For these types of strains (such as *Celeribacter halophilus*, *Roseobacter denitrificans*, *Roseovarius indicus*, *Ruegeria pomeroyi*, and *Sulfitobacter noctilucicola*), ammonium cross-feeding after methylamine degradation showed species-specific patterns, whereas bacterial GBT degradation always led to diatom growth. Overall, the degradation of DON compounds by the *Rhodobacteraceae* family and the subsequent cross-feeding of ammonium may represent a widespread, organism-specific, and regulated metabolic interaction for establishing and stabilizing associations with photoautotrophic diatoms in the oceans.

## Introduction

In the oceans, diatoms (Bacillariophyceae) are ubiquitous microalgae that are important in the biogeochemical cycles of carbon and nitrogen (Smetacek, 1999). In general, microalgae are surrounded by a boundary layer, such that mass transfer is diffusion controlled and not affected by convection; this so-called phycosphere is inhabited by heterotrophic bacteria that interact with the photoautotrophic microalgae in various ways, most of which are still not understood (Seymour et al., 2017). As previous studies have found that certain bacteria, namely, members of the *Alteromonadaceae*, *Bacteroidetes*, and *Rhodobacteraceae* families (Teeling et al., 2012; Goecke et al., 2013; Buchan et al., 2014), frequently co-occur with diatoms, specific metabolic interactions might be responsible for stabilizing these defined associations.

A major reason why heterotrophic bacteria establish transient and permanent interactions with diatoms is that diatoms perform autotrophic CO_2_ fixation, which causes them to exude dissolved organic carbon (DOC) (Amin et al., 2012) made up of various carbohydrates (Passow, 2002; Grossart et al., 2006, 2007; Molino and Wetherbee, 2008; Agogué et al., 2014; Klein et al., 2014; Buhmann et al., 2016; Doghri et al., 2017) as well as free amino acids and proteins (Gärdes et al., 2012; Paul et al., 2013; Amin et al., 2015; Durham et al., 2015; Stahl and Ullrich, 2016). In this DOC pool, heterotrophic bacteria also have access to compounds such as dimethylsulfoniopropionate (DMSP), dimethylsulfoxide, or taurine, which are produced and excreted by various phytoplankton species (Archer et al., 2011; Amin et al., 2015; Durham et al., 2015; Bullock et al., 2017). In return, the diatom-accompanying bacterial community can positively affect diatom growth and fitness (Amin et al., 2012). For example, bacteria provide diatoms and phytoplankton with growth promoters such as B vitamins and their precursors as well as indole-acetic acid (Bertrand et al., 2013; Amin et al., 2015; Wienhausen et al., 2017).

In the marine environment, however, nitrogen is often the limiting growth factor (Klawonn et al., 2019). In particular, as ammonium and nitrate are the essential nitrogen sources for diatoms, these compounds likely limit diatom growth throughout most of the year in open ocean habitats (Rogato et al., 2015). Diatoms can receive ammonium from nitrogen-fixing bacteria, but this process is dominated by cyanobacteria; the heterotrophic bacteria associated with diatoms usually cannot fix nitrogen (Berthelot et al., 2015).

This calls attention to dissolved organic nitrogen (DON) compounds which are released by many organisms in marine environments. For example, one study showed that by degrading DON, the bacterial metabolism can contribute ammonium and nitrate to microalgae (Klawonn et al., 2019). Yet, apart from common DON metabolites such as amino acids, which most heterotrophic bacteria can metabolize, there are many DON compounds than only few types of bacteria can degrade. Examples of such DON compounds are methylamines (MAs) and glycine betaine (GBT); these compounds may increase in concentration after upwelling events and can also be released by diatoms (Gibb et al., 1999; Keller et al., 1999a; Facchini et al., 2008; Müller et al., 2009).

Monomethylamine (MMA) is a ubiquitous nitrogen compound in marine habitats (Gibb et al., 1999; Lidbury et al., 2014), which is formed by enzymatic degradation of proteins and other organic nitrogen compounds (e.g., the osmolyte GBT); oceans also contain dimethylamine (DMA) and trimethylamine (TMA), but they are less abundant than MMA in the open waters (Gibb et al., 1999); in salt marshes and mud flats, DMA can be much higher (Mausz and Chen, 2019). Although these nitrogenous compounds are available, they cannot be utilized by diatoms (Balch, 1986). In contrast, many heterotrophic marine bacteria can use DON compounds and may liberate ammonium from them (Klawonn et al., 2019). For example, while methylotrophic bacteria do not degrade but rather assimilate the C_1_-compounds of MAs, non-methylotrophic bacteria can degrade these compounds, thereby releasing ammonium and conserving energy by catabolizing the C_1_ unit (Sun et al., 2011; Lidbury et al., 2015). In particular, members of the *Rhodobacteraceae* family can degrade TMA, DMA, and MMA by the consecutive action of TMA monooxygenase (Tmm), yielding trimethylamine-*N-*oxide (TMAO), TMAO demethylase (Tdm), DMA dehydrogenase (DmmDABC), and the *N*-methylglutamate (NMG) pathway (Chen et al., 2010; Latypova et al., 2010; Lidbury et al., 2014, 2017); to be rendered a suitable nitrogen source, the methylamines have to be degraded by the combined activity of all enzymes (see Supplementary Figure 1).

Recently, we showed that a member of the *Rhodobacteraceae* family, namely, the *Donghicola* sp. strain KarMa, can degrade MMA and subsequently release ammonium, which, in turn, can support the growth of the diatom *Phaeodactylum tricornutum* (Suleiman et al., 2016). In particular, we found that under photoautotrophic conditions, a coculture of strain KarMa and *P. tricornutum* can grow with MMA as the sole nitrogen source. It has not yet been shown whether DMA and TMA can also serve as nitrogen sources in such a mutualistic bacteria–diatom interaction. Further, since strain KarMA is a non-methylotrophic bacterium that cannot assimilate C_1_ compounds, in this coculture model system, the bacterial growth must be supported by a carbon source offered by the diatom; this carbon source is also still unknown.

The fact that methylamine degradation is widespread among diatom-associated bacteria from the *Rhodobacteraceae* family suggests that this metabolic trait may encourage specific groups of heterotrophic bacteria and photoautotrophic diatoms to form stable interactions. To investigate this hypothesis, we initiated our study with strain KarMa and *P. tricornutum* as a model system to explore the substrate spectrum of methylamines and, additionally, other relevant DON compounds (such as GBT) for this metabolic interaction. We were also interested which carbon sources might be provided by the diatom to the bacterium. Furthermore, we aimed at widening this analysis to different diatoms and different *Rhodobacteraceae* strains.

## Experimental Procedures

### Organisms and Growth Media

The diatoms *P. tricornutum* (strain UTEX 646) and *T. pseudonana* (CCMP1335) were kindly provided by Peter Kroth (Konstanz, Germany). The diatom *A. coffeaeformis* (CCAP1001/1) was obtained from the SAMS Research Ltd. (Scotland). All diatom strains were verified as axenic as described previously (Zecher et al., 2015). The marine bacterium *Donghicola* sp. strain KarMa was isolated and characterized in a previous study regarding its physiology (Suleiman et al., 2016) and its genome (Zecher et al., 2017). The type strains *Alteromonas macleodii* DSM6062, *Celeribacter halophilus* DSM26270, *Marinobacter adhaerens* DSM23420, *Roseobacter denitrificans* DSM7001, *Roseovarius indicus* DSM26383, *Ruegeria pomeroyi* DSS-3 (DSM15171), *Sulfitobacter noctilucicola* DSM101015, and *Sulfitobacter pseudonitzschiae* DSM26824 were obtained from the DSMZ (Germany). All microorganisms were cultivated in the marine mineral medium SW-f/2, which is based on the commercial Tropic Marine Salts mixture (Tropic Marine, Germany) in a final concentration of 3.32% (w/v) as described previously (Suleiman et al., 2016).

### Growth Experiments With Diatoms in Monocultures

Photoautotrophic growth of the three diatom species in monoculture was measured by chlorophyll fluorescence as described previously (Zecher et al., 2015). Pre- and main cultures were incubated in Erlenmeyer flasks at 21°C, 100 rpm (Orbital Shaker 3015, GFL, Germany) and a photon flux density of approximately 90 μE m^–2^ s^–1^ (light source: Lumilux Warmwhite, Osram, Germany) with a 14:10 h light/dark cycle in a light incubator (Phytobiochamber Model EGCS 701, EQUiTEC). Precultures of diatoms were inoculated in 10 ml SW-f/2 with either 2 mM ammonium or nitrate in 50 ml Erlenmeyer flasks from solid media and incubated for 4–7 days. Main cultures were inoculated from precultures to a chlorophyll concentration of 0.02 μg ml^–1^ with the respective nitrogen source in 30 ml SW-f/2 in 100 ml Erlenmeyer flasks, in 100 ml SW-f/2 in 250 ml Erlenmeyer flasks, or 1 ml SW-f/2 in 24-well plates (Nunclon^TM^ Delta Surface, Thermo Scientific, United States).

### Growth Experiments With Bacterial Strains in Monocultures

Growth experiments with all bacterial strains in monoculture were performed in different incubation vessels depending on the research question: either in 10 ml test tubes with 3 ml SW-f/2, in 24-well plates (Sarstedt, Germany) with 1 ml SW-f/2, or in 96-well plates (Sarstedt, Germany) with 200 μl SW-f/2. All incubations were performed at 30°C on a rotatory shaker at 200 rpm (Orbital Shaker 3015, GFL, Germany). Growth was measured by optical density at a wavelength of 600 nm (OD_600_) with a photometer (Spectrophotometer M107, Camspec, United Kingdom), by optical density at a wavelength of 595 nm (OD_595_) with a microplate reader (GENios^TM^ microplate reader, Tecan Group, Switzerland), or by colony forming units (CFUs) as described previously (Jagmann et al., 2010) by decimally diluting cultures in SW-f/2 medium without carbon and nitrogen sources and plating appropriate dilution steps on SW-f/2 plates containing 10 mM succinate and 2 mM MMA. Precultures were inoculated from solid media. Main cultures were inoculated from precultures to an OD_600_ of 0.01. Prior to inoculation of main cultures, appropriate volumes of the precultures were washed by centrifugation at 10,000 × *g* for 5 min at room temperature. Pre- and, unless otherwise stated, main cultures of *Donghicola* sp. strain KarMa were incubated with either 10 mM glucose or 20 mM succinate as carbon sources, depending on the research questions. Nitrogen sources were always added to a final concentration of 2 mM. For all other *Rhodobacteraceae* strains, pre- and, unless otherwise stated, main cultures were incubated with 0.2% (w/v) tryptone; these experiments were carried out in 24-well plates and included strain KarMa as a positive control, which was cultivated under the same conditions. All growth kinetics in mono- and cocultures were performed as biological duplicates with three technical replicates, as indicated in each legend.

### Substrate Spectrum of Carbon Utilization of Strain KarMa

Analysis of the substrate spectrum for different carbon sources of strain KarMa was performed in 96-well plates, as this experiment did not require sampling. For this, precultures of strain KarMa containing 2 mM ammonium and 10 mM glucose were used. The following carbon sources were tested (final concentrations in parentheses): *N*-acetylglucosamine (10 mM), arabinose (10 mM), arginine (5 mM), asparagine (5 mM), glycine betaine (GBT, 10 mM), putrescine (1 mM), spermidine (1 mM), citric acid (10 mM), dimethyl sulfoniopropionate (DMSP, 10 mM), eicosapentaenoic acid (EPA, 1 mM), fructose (10 mM), galactose (10 mM), galacturonic acid (10 mM), glucoronic acid (10 mM), glucose (10 mM), glycine (5 mM), histidine (5 mM), hypoxanthine (10 mM), isocitric acid (10 mM), isoleucine (5 mM), α-ketoglutaric acid (10 mM), lactose (10 mM), lysine (5 mM), malic acid (10 mM), maltose (10 mM), mannose (10 mM), oxalic acid (10 mM), acetate (10 mM), propionate (10 mM), formate (10 mM), glycolate (10 mM), glyoxylate (10 mM), succinate (10 mM), sucrose (10 mM), taurine (10 mM), tryptophan (5 mM), urea (5 mM), valine (5 mM), and xylose (10 mM). Due to the low pK_*a*_ or pK_*b*_ values of several carbon sources, a neutralization of the stock solutions to pH ∼7 using NaOH or HCl, respectively, was necessary. Carbon concentrations were not normalized, as these experiments were aimed at screening substrates. To evaluate the degree of growth, the growth of the cultures was compared to a positive control, containing 10 mM as the carbon source, and to a negative control without any organic carbon source.

In addition to the experiments described above, it was tested whether formate, glycolate, glyoxylate, and taurine, which all failed to support growth as sole substrates, could increase growth yields of strain KarMa when supplied in addition to succinate as a utilizable carbon source. These experiments were performed in 10 ml glass test tubes. Precultures of strain KarMa contained 2 mM ammonium and 2 mM succinate. Further, 20 mM formate or 10 mM glycolate, glyoxylate, or taurine was added to the precultures to ensure induction of possible dissimilatory pathways. Main cultures were inoculated in glass test tubes in SW-f/2 medium containing 2 mM ammonium and 2 mM succinate with 20 mM formate or 10 mM glycolate, glyoxylate, or taurine, respectively.

### Substrate Spectrum of Nitrogen Utilization of Strain KarMa

Analysis of the substrate spectrum of different nitrogen sources for strain KarMa was performed in 10 ml glass tubes. Precultures of strain KarMa cells were cultivated with 10 mM glucose and the respective nitrogen source that was to be used in further cultivation experiments. The following nitrogen sources were applied at a concentration of 400 μM: ammonium, nitrate, nitrite, monomethylamine (MMA), dimethylamine (DMA), trimethylamine (TMA), glycine betaine (GBT), taurine, putrescine, spermidine, and urea. Organic nitrogen concentrations were not normalized, as these experiments served to screen substrates. Main cultures for the growth experiments were inoculated in glass test tubes and cultivated at 30°C at 200 rpm for 3 days.

### Growth Experiments in Cocultures

Cocultures of the three diatoms with strain KarMa were set up in 100 ml Erlenmeyer flasks containing 50 ml SW-f/2; as these experiments involved regular sampling, a larger culture volume was required. Cocultures of the other *Rhodobacteraceae* strains were performed in 24-well plates (Nunclon^TM^ Delta Surface, Thermo Scientific, United States) containing 1 ml SW-f/2. The respective nitrogen sources were added with a final concentration of 2 mM to all cocultures. For inoculating cocultures, main cultures of the individual microorganisms were set up as described above. Appropriate volumes of the bacterial main cultures and of the diatom main cultures were added to the coculture medium to yield a starting OD_600_ of 0.01 for the bacteria and a chlorophyll concentration of 0.02 μg ml^–1^ for the diatoms. Diatom growth in cocultures was measured by chlorophyll fluorescence using aliquots taken at regular intervals from the growing cultures. For measuring chlorophyll fluorescence in Erlenmeyer flasks, 1 ml aliquots were transferred to a microtiter plate, which was placed into an imaging device as described previously (Zecher et al., 2015). For measuring chlorophyll fluorescence in cocultures in 24-well plates, the plates were directly placed into the imaging device. Bacterial growth in coculture was determined by CFU, as described above for monocultures.

### Determination of Ammonium, MMA, and DMA Concentrations

Ammonium, MMA, and DMA were determined by derivatization of the amino group with diethylethoxymethylenemalonate (DEEMM) followed by high-performance liquid chromatography (HPLC) analysis. For this, aliquots from culture supernatants were centrifuged at 16,000 × g at room temperature for 15 min to remove the cells. The supernatant was either stored at −20°C until analysis or directly derivatized with DEEMM and separated by HPLC with a C_18_ reversed phase column at 40°C (Vertex Plus C18 reversed phase, 250 × 3 mm, Knauer, Germany) as described previously (Suleiman et al., 2016). The linear detection range was determined to be between 50 μM and 2 mM.

### *In silico* Analysis of Bacterial Genes Putatively Involved in the Degradation of Methylamine and GBT

Genes for the degradation of MMA and GBT were identified previously in the genome of strain KarMa (Zecher et al., 2017). Genes for the degradation of DMA and TMA were identified in the genome of strain KarMa by basic local alignment search tool (BLAST) analysis [National Center for Biotechnology Information (NCBI) BLASTp analysis with default settings; Altschul et al., 1990] using sequences of the TMA monooxygenase (*tmm*), the trimethylamine-*N*-oxide demethylase (*tdm*), and the DMA monooxygenase (*dmmCBAD*) from *R. pomeroyi* DSS-3 (DSM15171) as query sequences because the respective function of these genes had been verified experimentally in this bacterium (Lidbury et al., 2015; Lidbury et al., 2017). The best BLAST hit for each protein was assigned the respective function in strain KarMa, and all hits had e values of 1e^–73^ or below, at least 49% identity and at least 93% query coverage (Supplementary Table 1). Reciprocal BLAST analysis was performed to verify that these genes are orthologs in *R. pomeroyi* DSS-3. Representative genes for the degradation of MMA, GBT, DMA, and TMA within the family of the *Rhodobacteraceae* (Boratyn et al., 2012) were identified by Domain Enhanced Lookup Time Accelerated (DELTA)-BLAST analysis (NCBI DELTA BLASTp analysis with default settings) using the protein sequences of Tmm (SCM66286.1), Tdm (SCM66287.1), DmmD (SCM66269.1), GMAS (SCM66280.1), MgsC (SCM66281.1), MauB (SCM67379.1), and Bhmt1 (SCM66178.1) from strain KarMa as query sequences. Based on this analysis, six *Rhodobacteraceae* strains (*C. halophilus* DSM26270, *R. denitrificans* DSM7001, *R. indicus* DSM26383, *R. pomeroyi* DSS-3, *S. noctilucicola* DSM101015, *S. pseudonitzschiae* DSM26824), which are commercially available as type strains as well as two representatives of the marine *Gammaproteobacteria* of the *Alteromonadaceae* family (*A. macleodii* DSM6062 and *M. adhaerens* DSM23420) known to interact with diatoms (Gärdes et al., 2012; Diner et al., 2016) were chosen for a more thorough analysis of the gene synteny of the degradation pathways. In these organisms, MMA, GBT, DMA, and TMA degradative gene clusters were predicted and annotated using BLASTp analysis (minimum e value e^–50^) with the respective protein sequences encoded in the predicted gene clusters of strain KarMa as queries.

## Results

### Substrate Spectrum of *Donghicola* sp. Strain KarMa in Monoculture

**FIGURE 1:**
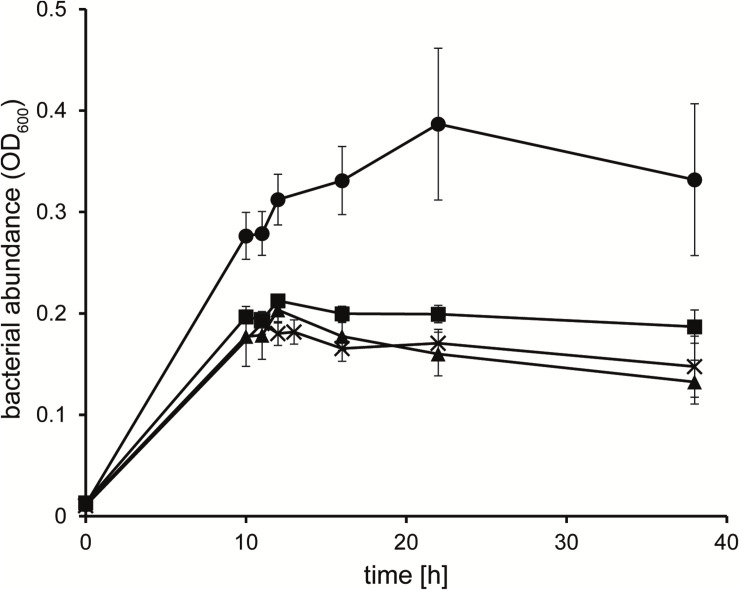
Growth of *Donghicola* sp. strain KarMa with 2 mM succinate in the absence (◼) of other organic carbon sources and in the presence of 10 mM glycolate (•), 10 mM glyoxylate (▲), and 20 mM formate (X). Error bars indicate standard deviation (*n* = 3).

To identify the potential carbon and nitrogen sources that the heterotrophic strain KarMa might receive from the photoautotrophic diatom *P. tricornutum*, in bacterial monocultures, we tested as growth substrates various carbohydrates, carboxylic acids, as well as amino acids and further DON compounds that may be actively or passively released by diatoms (Table 1). Except for *N*-acetylglucosamine, strain KarMa was able to use all carbohydrates and most of the amino acids as carbon and energy sources. Regarding carboxylic acids occurring as intermediates of the tricarboxylic acid cycle (TCA), all except for iso-citrate could be used. The C_2_ and C_1_ carboxylic acids glycolate, glyoxylate, and formate could not be used; to test if they could be dissimilated by strain KarMa, cells were incubated with low amounts of succinate (2 mM) as carbon and energy source with addition of formate, glycolate, or glyoxylate (Figure 1). The addition of glycolate caused a doubling of OD_600_ compared to succinate-only control cultures, while the addition of formate and glyoxylate had no effect. The common algal exudates GBT and DMSP could also be used as growth substrates, but the polyunsaturated fatty acid EPA, which is produced by diatoms and other microalgae (Good et al., 2015; Shi et al., 2018), could apparently not be used as a growth substrate and even had negative effects on the growth of strain KarMa with glucose (not shown). The polyamines putrescine and spermidine are also diatom-related DON compounds, as they occur in the ornithine–urea cycle and are involved in precipitation of the silicified cell wall (Kröger et al., 2000; Allen et al., 2006; Sumper and Lehmann, 2006). These polyamines as well as DMA and TMA and also taurine could not be used as carbon sources. The substrate spectrum of strain KarMa revealed that this bacterium was able to use for growth most of the selected compounds that have been shown to be released by diatoms. Except for taurine, all other DON compounds (DMA, TMA, GBT, and the polyamines) could be used as sole source of nitrogen (Table 1). In addition, strain KarMa was also able to use urea, nitrate, and nitrite as nitrogen sources.

**TABLE 1:** Utilization of organic compounds by strain KarMa in monoculture experiments.

Carbohydrates	A	B	TCA intermediates	A	B
Glucose	+	ia.	Citrate	+	ia.
Fructose	+	ia.	Iso-citrate	-	ia.
Mannose	+	ia.	α-ketoglutarate	+	ia.
Galactose	+	ia.	Succinate	+	ia.
Arabinose	+	ia.	Malate	+	ia.
Xylose	+	ia.	Oxaloacetate	+	ia.
			
Maltose	+	ia.	**Additional metabolites**	**A**	**B**
			
Lactose	+	ia.	Formate	-	ia.
Sucrose	+	ia.	Monomethylamine	-	+
*N*-acetylglucosamine	-	n.a.	Dimethylamine	-	+
Galacturonic acid	+	ia.	Trimethylamine	-	+
Glucuronic acid	+	ia.	Urea	-	+
			
**Amino acids and mixtures**	**A**	**B**	Acetate	+	ia.
			
Arginine	+	n.a.	Glycolate	-	ia.
Histidine	+	n.a.	Glyoxylate	-	ia.
Lysine	+	n.a.	Propionate	+	ia.
Asparagine	+	n.a.	Glycine betaine	+	+
Glycine	+	n.a.	Putrescine	-	+
Tryptophan	-	n.a.	Spermidine	-	+
Valine	-	n.a.	Taurine	-	-
Isoleucine	+	n.a.	Dimethyl sulfoniopropionate	+	ia.
Tryptone	+	+	Eicosapentaenoic acid	+	ia.

*Cells were incubated for 3 days; the organic compounds were either supplied as (A) carbon and energy sources or (B) as nitrogen sources with 10 mM glucose as carbon source; + indicates an increase in optical density (OD_600_ > 0.1; this value was never exceeded in media without organic substrates); - indicates no increase in optical density; n.a. indicates “not analyzed”; ia. indicates “inapplicable.”*

### Cocultivation of Strain KarMa and *P. tricornutum* With Various Nitrogen Sources

**FIGURE 2:**
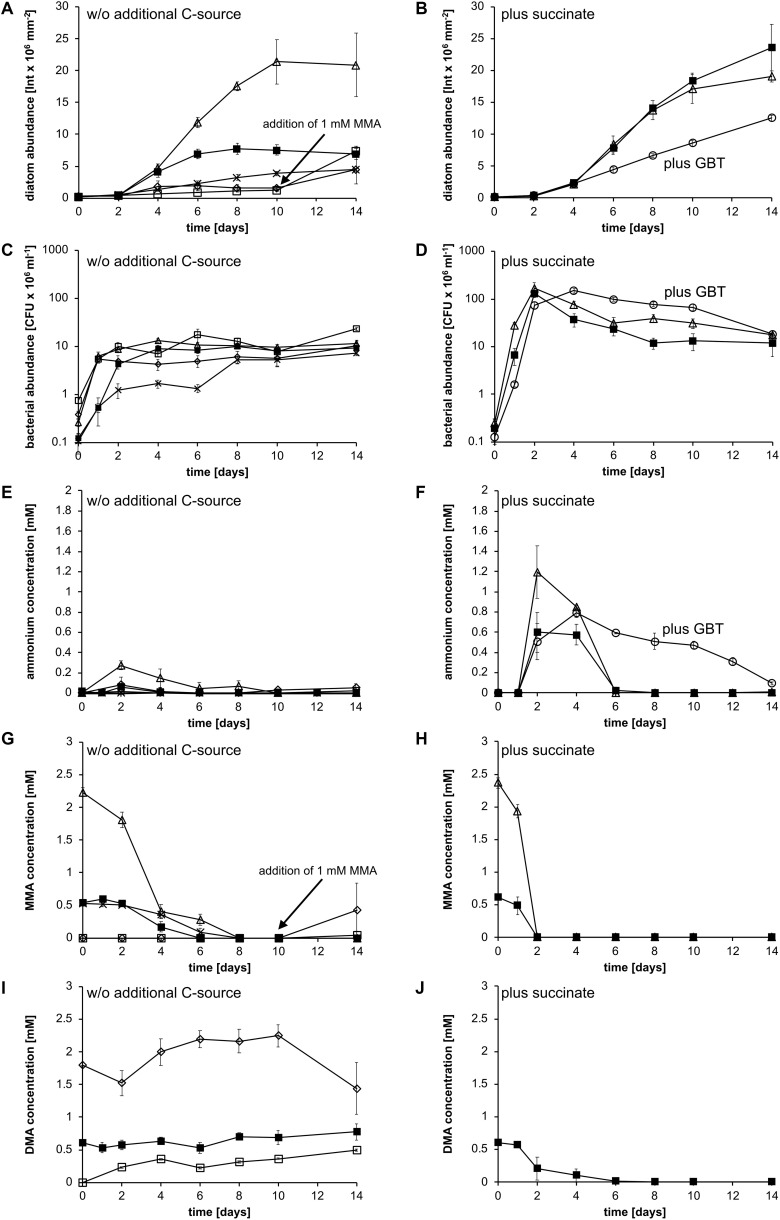
Growth of *P. tricornutum* and in cocultures with *Donghicola* sp. strain KarMa under photoautotrophic conditions **(A,C,E)** in the absence and **(B,D,F)** in the presence of additional carbon and energy sources. Algal and bacterial abundance were determined by chlorophyll fluorescence and CFU counts, respectively, the methylamine mixture contained 0.66 mM of each monomethylamine (MMA), dimethylamine (DMA), and trimethylamine (TMA). **(A)** Abundance of *P. tricornutum* with 2 mM MMA (△), DMA (⋄), TMA (◻), the methylamine mixture (◼), and 0.66 mM MMA (×) as nitrogen sources. **(B)** Abundance of *P. tricornutum* with 2 mM succinate and either 2 mM MMA (△) or the methylamine mixture (◼) or with 2 mM GBT (○) as nitrogen and carbon source. **(C)** Abundance of strain KarMa with 2 mM MMA (△), DMA (⋄), TMA (◻), the methylamine mixture (◼), and 0.66 mM MMA (×) as nitrogen sources. **(D)** Abundance of strain KarMa with 2 mM succinate and either 2 mM MMA (△) or the methylamine mixture (◼) or with 2 mM GBT (○) as nitrogen and carbon source. **(E)** Ammonium concentrations of cocultures with 2 mM MMA (△), DMA (⋄), TMA (◻), the methylamine mixture (◼), and 0.66 mM MMA (×) as nitrogen sources. **(F)** Ammonium concentrations of cocultures with 2 mM succinate and 2 mM MMA (△) or methylamine mixture (◼) as nitrogen sources and with 2 mM GBT (○) as carbon and nitrogen source. **(G)** MMA concentrations of cocultures with 2 mM nitrogen sources in form of MMA (△), DMA (⋄), TMA (◻), and methylamine mixture (◼) as well as 0.66 mM MMA (×). **(H)** MMA concentrations of cocultures with 2 mM succinate and either 2 mM MMA (△) or 2 mM methylamine mixture (◼) or with 2 mM GBT (○) as nitrogen and carbon source. **(I)** DMA concentrations of cocultures with 2 mM nitrogen sources in form of DMA (⋄), TMA (◻), and methylamine mixture (◼). **(J)** DMA concentrations of cocultures with 2 mM succinate and 2 mM methylamine mixture (◼). Error bars indicate standard deviation for diatom abundance (*n* = 3) and standard error of the mean for bacterial abundance (*n* = 3).

In the next step, we investigated whether those organic nitrogen sources that could be utilized by strain KarMa and that are also known to occur as DON in the marine water column can support the growth of *P. tricornutum* when offered as the sole nitrogen source in coculture with strain KarMa. In all cultivations, except for the control with 0.66 mM MMA, the total organic nitrogen concentration was adjusted to 2 mM. Methylamine mixtures contained equal amounts of MMA, DMA, and TMA. Regarding the methylamines, cocultures with MMA reached the highest diatom abundance compared to cocultures containing either DMA or TMA; in those cultures, almost no diatom growth was observed (Figure 2A). When these cultures were supplied with 1 mM MMA after day 10, a clearly observable diatom growth occurred, indicating that DMA and TMA had no general inhibiting effect on diatom growth. The second highest diatom abundance was achieved for mixtures of MMA, DMA, and TMA adding up to an organic nitrogen concentration of 2 mM. Cocultures with 0.66 mM MMA reached a lower diatom abundance than those containing 2 mM but a higher abundance than the DMA and TMA cocultures prior to MMA addition. Bacterial abundance of all cultures increased in the first days and remained relatively constant throughout the cultivation, except for cocultures containing 0.66 mM MMA (Figure 2C). However, as strain KarMa tends to form cell chains in cocultures with the diatoms, as was previously observed in cocultures with *P. tricornutum* (Suleiman et al., 2016), the bacterial abundance determined by CFUs is likely to be an underestimate.

Ammonium concentrations in the supernatant of cocultures containing 2 mM MMA increased from the start until day 2 and subsequently decreased below the detection limit at day 10 (Figure 2E). For cocultures with DMA, TMA, or the methylamine mixture, only small amounts of ammonium were detected at day 2; at all other sampling points, ammonium concentrations were close to or below the detection limit. For the cocultures containing 2 mM MMA, MMA concentrations decreased from the start of the experiment until day 8, when they were below the detection limit (Figure 2G). For cocultures containing 0.66 mM MMA or the methylamine mixtures, MMA concentrations decreased more slowly and were below the detection limit at days 8 and 6, respectively. For cocultures containing DMA and TMA, MMA was only observed after the addition of 1 mM MMA at day 10 (Figure 2G). For DMA-containing cocultures, the DMA concentration was relatively constant until the addition of MMA at day 10 (Figure 2I); at 14 days and after the addition of MMA, a slight decrease in DMA concentration was observed. For cocultures containing TMA, an increase in DMA concentration was observed throughout the experiment, indicating that strain KarMa could transform TMA into DMA (Figure 2I). For cocultures containing the methylamine mixtures, the DMA concentration remained relatively constant throughout the experiment. Similar to in *P. tricornutum* monoculture experiments, where *P. tricornutum* could not use the polyamines putrescine and spermidine, adding putrescine and spermidine to cocultures of *P. tricornutum* and strain KarMa did not lead to an increase in diatom abundance (Supplementary Figure 2). Apparently, MMA was the best organic nitrogen source for *P. tricornutum* upon its degradation by strain KarMa. Finally, we examined whether urea that originates from the ornithine–urea cycle can serve as a nitrogen storage compound in diatoms, so it was tested as the sole nitrogen source in cocultures (Nunn et al., 2009; Allen et al., 2011; Bender et al., 2012). In mono- and cocultures that contained urea, the diatom abundances increased similarly (Supplementary Figure 2), which supports the idea that *P. tricornutum* possesses its own urease (Weyman et al., 2015).

### Cocultivation of Strain KarMa and *P. tricornutum* With MMA in the Presence of Additional Carbon and Energy Sources

For characterizing the process of ammonium cross-feeding from strain KarMa to diatoms on the physiological level, we set up cocultures of strain KarMa with *P. tricornutum* under different conditions. In the first step, we investigated whether supplying an additional algae-independent carbon and energy source for strain KarMa would affect ammonium cross-feeding in cocultures with methylamines as the sole nitrogen source. Supply of 2 mM succinate with MMA as the nitrogen source did not influence growth of *P. tricornutum* (Figure 2B in comparison to Figure 2A). In contrast, when 2 mM of succinate was supplied along with the methylamine mixture, this strongly enhanced the growth of *P. tricornutum* (Figure 2B in comparison to Figure 2A); bacterial growth was generally accelerated upon supply of succinate compared to purely photoautotrophic conditions (Figure 2C compared to Figure 2D). Supply of succinate also led to a higher transient accumulation of ammonium compared to cocultures under purely photoautotrophic conditions, especially with the methylamine mixture (Figure 2F compared to Figure 2E). In addition, MMA degradation was much faster in cocultures supplied with succinate (Figures 2G,H), and degradation of DMA in the coculture with the methylamine mixture was only observed in the succinate-supplied cocultures (Figures 2I,J). As we found that strain KarMa could transform TMA into DMA in the coculture in the absence of succinate (Figure 2I), the addition of succinate apparently enhanced the utilization of DMA and TMA compared to purely photoautotrophic conditions.

In a further experiment, we added GBT as an additional carbon and energy source. As strain KarMa was also able to use GBT as a nitrogen source while *P. tricornutum* was not (Supplementary Figure 3), we omitted MMA from these cocultures to investigate whether GBT is also a potential substrate for ammonium cross-feeding. Compared to MMA-containing cocultures with or without succinate, *P. tricornutum* grew much slower in GBT-containing cocultures (Figure 2B compared to Figure 2A); in contrast, bacterial growth was accelerated (Figure 2D). For GBT-containing cocultures, we observed an increase in ammonium until day 4, which was followed by a slow decrease.

### Cocultivation of Strain KarMa and *P. tricornutum* With MMA in the Presence of Nitrate

**FIGURE 3:**
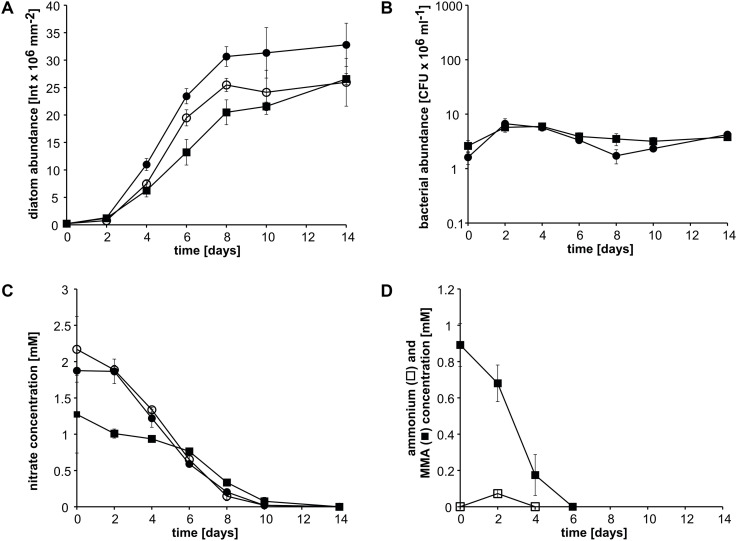
Growth of *P. tricornutum* monocultures and in cocultures with *Donghicola* sp. strain KarMa with 2 mM nitrate (•) or 1 mM nitrate plus 1 mM monomethylamine (MMA) (◼) compared to its growth in monocultures with 2 mM nitrate (○) under photoautotrophic growth conditions. Algal and bacterial abundance were determined by chlorophyll fluorescence and CFU counts, respectively. **(A)** Abundance of *P. tricornutum*. **(B)** Abundance of strain KarMa. **(C)** Nitrate concentrations within the cocultures shown in **(A)**. **(D)** Concentrations of ammonium (◻) and MMA (◼) of the cocultures with MMA and nitrate. Error bars indicate standard deviation for diatom abundance (*n* = 3) and standard error of the mean for bacterial abundance (*n* = 3).

In the next step, we investigated whether an additional nitrogen source for *P. tricornutum* would affect ammonium cross-feeding from MMA degradation. In cocultures with nitrate, *P. tricornutum* reached higher abundances than in cocultures with nitrate plus MMA and also than in monoculture with nitrate (Figure 3A). Bacterial abundances were always below 10^7^ CFU ml^–1^ in nitrate-containing cocultures, whereas those with MMA as the sole nitrogen source were at or above this value (Figure 3B compared to Figure 2C). Nitrate concentrations of nitrate-containing pure and cocultures decreased steadily and were nearly completely depleted at day 10 (Figure 3C). Nitrate concentrations of cocultures containing both MMA and nitrate decreased slowly until day 6; afterward, a faster decrease was observed until day 10. The concentration of MMA within the MMA-plus nitrate-containing cocultures decreased steadily and was below the detection limit at day 6, whereas ammonium was only detectable at day 2 (Figure 3D). These results show that nitrate did not affect MMA degradation and was, apparently, used concomitantly with ammonium.

### Cocultivation of Strain KarMa With *Amphora coffeaeformis* or *Thalassiosira pseudonana*

**FIGURE 4:**
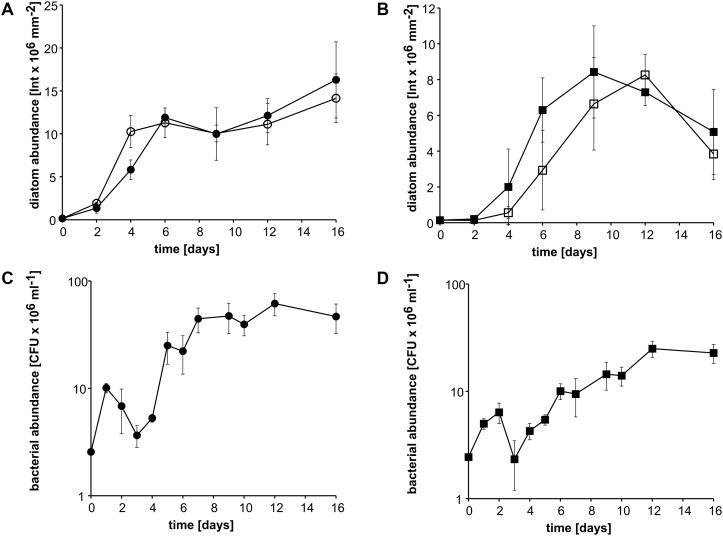
Growth of the diatoms *A. coffeaeformis* and *T. pseudonana* in cocultures with *Donghicola* sp. strain KarMa under photoautotrophic conditions. Algal and bacterial abundance were determined by chlorophyll fluorescence and CFU counts, respectively. **(A)** Abundance of *A. coffeaeformis* in cocultures with strain KarMa with 2 mM monomethylamine (MMA) (•) compared to monocultures with 2 mM ammonium (•) as nitrogen sources. **(B)** Abundance of *T. pseudonana* in cocultures with strain KarMa with 2 mM MMA (◼) compared to monocultures with 2 mM ammonium (◻) as nitrogen sources. **(C)** Abundance of strain KarMa in cocultures with *A. coffeaeformis* (•). **(D)** Abundance of strain KarMa in cocultures with *T. pseudonana* (◼). Error bars indicate standard deviation for diatom abundance (*n* = 3) and standard error of the mean for bacterial abundance (*n* = 3).

To investigate whether diatoms other than *P. tricornutum* also grow in coculture with ammonium derived from bacterial MMA cleavage, cocultures of the diatoms *Amphora coffeaeformis* or *Thalassiosira pseudonana* with *Donghicola* sp. strain KarMa were incubated with 2 mM MMA as the sole nitrogen source. Growth kinetics of *A. coffeaeformis* in cocultures with strain KarMa did not differ from growth kinetics in monocultures with 2 mM ammonium (Figure 4A). For *T. pseudonana*, growth kinetics were also very similar in coculture with strain KarMa compared to the monoculture with ammonium as the nitrogen source (Figure 4B). In both cocultures, the growth of strain KarMa was similar (Figures 4C,D). In monoculture, both diatoms were not able to grow with MMA as the sole nitrogen source (Supplementary Figure 4). Overall, these results indicate that interkingdom cross-feeding of ammonium via MMA degradation by strain KarMa was not restricted to the diatom *P. tricornutum*.

### Bioinformatic Analysis of Methylamine and GBT Degradation in Members of the *Rhodobacteraceae*

**FIGURE 5:**
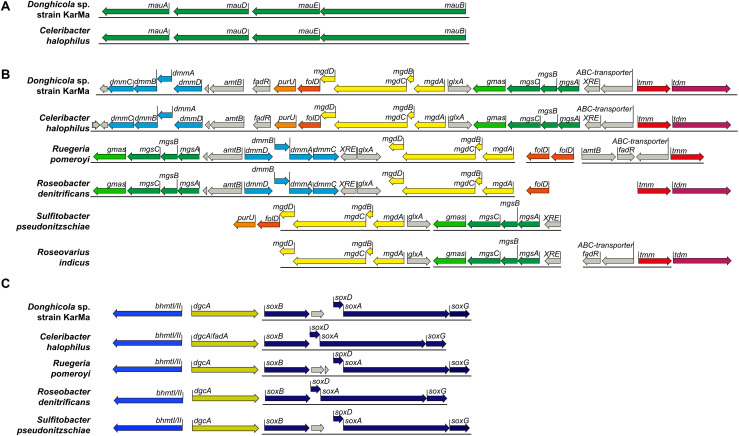
Overview of gene clusters encoding metabolic pathways for the degradation of methylamines and of glycine betaine (GBT) in selected marine bacteria within the family of the *Rhodobacteraceae*. The genes encode putative enzymes for metabolic pathways for **(A)** monomethylamine (MMA) degradation *via* the periplasmatic methylamine dehydrogenase; **(B)** MMA degradation via the *N*-methylglutamate (NMG) pathway and DMA and TMA degradation; **(C)** GBT degradation. Solid lines below genes indicate their adjacent position in the respective genomes. In particular, the genes encode enzymes for the following functions: **(A)** methylamine dehydrogenase encoded by *mauAB* with *mauD* and *mauE* for protein assembly. **(B)** TMA is oxidized by the TMA monooxygenase (*tmm*) to trimethylamine-*N-*oxide (TMAO) and further to DMA *via* the trimethylamine-*N*-oxide demethylase (*tdm*). DMA is further degraded to MMA by the DMA monooxygenase (*dmmCBAD*). MMA may be degraded by the NMG pathway encoded by gamma-glutamylmethylamide synthase (*gmas*), *N*-methylglutamate synthase (*mgsCBA*), and the *N*-methylglutamate dehydrogenase (*mgdDCBA*). The methyl groups from the consecutive demethylation may not be released as formaldehyde but may be bound to THF processed by the gene products of *folD* and *purU*. Several other genes (marked in gray) repeatedly co-occurring within the TMA/DMA/MMA degradation cluster with high similarity to strain KarMa genes are marked as well: *amt*, ammonium transporter; *fadR*, transcriptional regulator FadR family; *glxA*, transcriptional regulator GlxA family; *XRE*, transcriptional regulator XRE family; *ABC transporter*, ABC transporter substrate binding-protein. Gene nomenclature was chosen according to Latypova et al. (2010) and Lidbury et al. (2017). **(C)** GBT degradation *via* betaine homocysteine S-methyltransferase (*bhmtI*), dimethylglycine demethylase (*dgcA*), and sarcosine dehydrogenase (*soxBDAG*). Genes from *M. adhaerens* and *A. macleodii* as representatives of the *Alteromonadaceae* were included into this part of the genomic comparison.

As the ability to degrade methylamines is speculated to be common among *Rhodobacteraceae* (Lidbury et al., 2017), we wanted to investigate whether the ammonium cross-feeding from methylamine degradation is also possible with other members of the marine *Rhodobacteraceae*. This investigation was initiated by a bioinformatic analysis as described in section “Experimental Procedures.” Regarding the degradation of MMA, only *C. halophilus* contained both the *mau* gene cluster and the genes for the NMG pathway in a very similar arrangement as strain KarMa (Figures 5A,B); the other *Rhodobacteraceae* strains harbored only the NMG pathway. Regarding the degradation of DMA and TMA, in the genome of strain KarMa, the required genes were found in a cluster on the plasmid A (Figure 5B); again, this gene arrangement was very similar to that in *C. halophilus*, and similar genomic arrangements for DMA and TMA utilization were also found in *R. pomeroyi* and *R. denitrificans*. For *R. indicus*, putative MMA and TMA degradation genes were identified, whereas for *S. pseudonitzschiae*, only genes for MMA degradation were predicted. Genes for the degradation of all methylamines were all found to be in close proximity to each other. We also identified additional genes that colocalize with the putative degradation genes, namely, genes that encode two putative transporter subunits as well as several transcriptional regulators (Figure 5B).

As our coculture experiments indicated that GBT could also serve as substrate from ammonium cross-feeding from bacteria to diatoms, genes encoding enzymes for its degradation (Meskys et al., 2001; Wargo, 2013) were also included in the bioinformatic analysis. These comprised genes encoding betaine homocysteine S-methyltransferase (*bhmt1*), dimethylglycine demethylase (*dgcA*), and sarcosine oxidase (*soxBDAG*), which were identified in the genome of strain KarMa. All the above-mentioned *Rhodobacteraceae* strains harbored genes for Bhmt1, DgcA, and SoxBDAG (Figure 5C). In A. *macleodii* and *M. adhaerens*, all genes for methylamine degradation as well as for SoxBDAG were not detected, while only genes for Bhmt1 and DgcA were present (not shown).

### Methylamine and GBT Utilization of *Rhodobacteraceae* Type Strains in Monoculture and in Coculture With *P. tricornutum*

**FIGURE 6:**
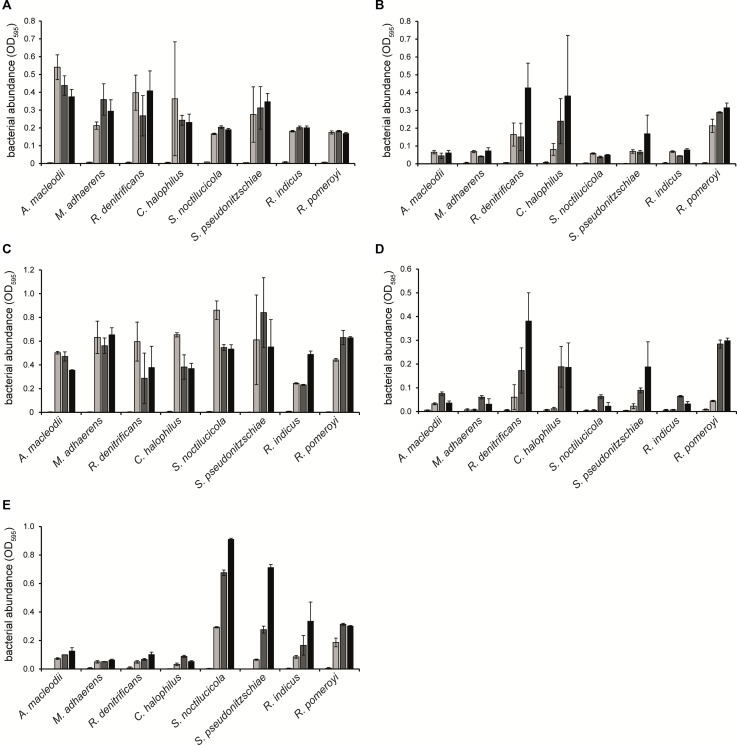
Growth of *Rhodobacteraceae* and *Alteromonadaceae* type strains in monocultures with **(A)** 0.1% (w/v) tryptone; **(B)** 2 mM monomethylamine (MMA) and 10 mM glucose; **(C)** 2 mM MMA, 10 mM glucose, and 0.1% (w/v) tryptone; **(D)** 2 mM methylamine mixture and 10 mM glucose; and **(E)** 2 mM GBT and 10 mM glucose, all in 24-well plates. Growth was followed for 3 days (day 0, white; day 1, light gray; day 2, dark gray; day 3, black). Error bars indicate standard deviation (*n* = 3).

The six additional *Rhodobacteraceae*-type strains as well as *Alteromonadaceae*-type strains *A. macleodii* and *M. adhaerens* were analyzed in mono- and coculture experiments for their ability to degrade methylamines or GBT and to potentially support growth of *P. tricornutum*, as a model organism for diatoms. All strains grew similarly with tryptone in the absence and presence of the methylamine mixture, suggesting that methylamines do not have toxic effects on growth (Figures 6A,C). For classifying whether a strain could grow with methylamines as the nitrogen source, we considered that the very slight increase in OD_600_ (<0.1) seen in the *Alteromonadaceae* strains (Figures 6B,D,E), which lack the genes for methylamine and GBT degradation, represented no growth. With glucose as the carbon and energy source, *R. denitrificans*, *C. halophilus*, *S. noctilucicola*, and *R. pomeroyi* were able to grow with MMA or the methylamine mixture as nitrogen sources (Figures 6B,D). In contrast, *S. pseudonitzschiae* and *R. indicus* did not grow under these conditions. With glucose as the carbon and energy source and GBT as the nitrogen source, *S. pseudonitzschiae*, *S. noctilucicola*, *R. indicus*, and *R. pomeroyi* were able to grow, while *R. denitrificans* and *C. halophilus* were not (Figure 6E). Considering that all *Rhodobacteraceae* strains harbored the genes for MMA and GBT utilization, the genomic information could not be used in all cases to predict whether the strain could use these compounds.

**FIGURE 7:**
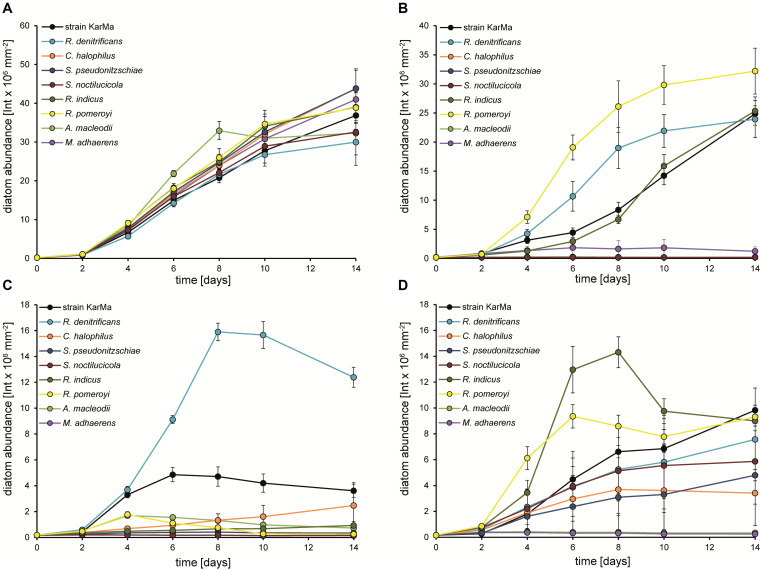
Growth of *P. tricornutum* in cocultures with strain KarMa in comparison to various type strains of *Rhodobacteraceae* and to *A. macleodii* with **(A)** 2 mM ammonium, **(B)** 2 mM monomethylamine (MMA), **(C)** 2 mM methylamine mixture, or **(D)** 2 mM glycine betaine (GBT) as the sole nitrogen sources. Error bars indicate standard deviation for diatom abundance determined by chlorophyll fluorescence (*n* = 3).

Cocultures of *P. tricornutum* and strain KarMa were used as a reference culture for all tested nitrogen sources (Figure 7). Diatom abundance increased similarly in all cocultures with ammonium (Figure 7A). With MMA as the sole nitrogen source, an increase in diatom abundance was observed for cocultures with *R. denitrificans*, *R. indicus*, and *R. pomeroyi* (Figure 7B). Interestingly, the growth of *R. indicus* in monoculture with glucose and MMA was weak, and *C. halophilus*, which was able to grow with MMA as the nitrogen source in monoculture, did not support the growth of diatoms in coculture (Figure 6B). With the methylamine mixture as the nitrogen source, *R. denitrificans* had the highest increase in diatom abundance (Figure 7C), which corresponded to its growth in monoculture (Figure 6D). For *C. halophilus* cocultures with the methylamine mixture, we observed a slow but constant increase in diatom abundance (Figure 7C), which was also consistent with its growth in monoculture (Figure 6D). All other cocultures with the methylamine mixture showed no or very little diatom growth. In cocultures with the *Rhodobacteraceae*-type strains containing GBT as the nitrogen source, diatom abundance increased in all of them but to different extents (Figure 7D). Interestingly, *R. denitrificans* and *C. halophilus* did not grow with GBT as the nitrogen source in monocultures (Figure 6E). The *Alteromonadaceae*-type strains did not lead to an increase in diatom abundance with either methylamines or GBT as the sole nitrogen source, and they did not inhibit diatom growth when ammonium was used as the nitrogen source (Figures 7A–D).

## Discussion

The goal of this study was to investigate whether ammonium cross-feeding from MMA-degrading *Rhodobacteraceae* to diatoms, which we had originally shown for *Donghicola* sp. strain KarMa and *P. tricornutum* (Suleiman et al., 2016), has a broader ecological significance for overcoming the frequent nitrogen limitation for phytoplankton in the open ocean (Rogato et al., 2015). Based on a cultivation-based physiological analysis, we obtained evidence that ammonium cross-feeding from DON-degrading *Rhodobacteraceae* to diatoms could indeed be a widespread interaction in the phycosphere in marine habitats because we were able to extend this concept to additional diatoms, to additional Rhodobacteriales and to additional DON compounds. In particular, strain KarMa appears to be very well adapted to this ecological niche in a species-independent way, as it supported the growth of two additional diatoms, namely, *A. coffeaeformis* and *T. pseudonana*, by providing ammonium from MMA degradation. Remarkably, diatom growth in the MMA-containing cocultures did not differ from diatom growth in ammonium-containing monocultures, indicating that MMA cleavage kinetics were suitably coordinated with diatom growth kinetics. Importantly, strain KarMa’s diatom-supporting properties certainly depend on its ability to use a variety of carbon sources that are typically exuded by diatoms, such as selected carbohydrates, glycolate, and GBT. Further, ammonium cross-feeding was even enhanced by the presence of additional carbon sources: The addition of succinate in cocultures with *P. tricornutum*-enabled strain KarMa to utilize DMA and TMA.

The degradation of TMA and DMA requires the bacteria to perform additional steps to convert these compounds into MMA, which is then split into ammonium and formaldehyde by two different enzyme systems in *Rhodobacteraceae* (Latypova et al., 2010). According to strain KarMa’s genetic repertoire, these oxidative demethylations of the nitrogen atom are catalyzed by two monooxygenases (Tmm and DmmABCD) and a methyltransferase (Tdm). As strain KarMa could use TMA and DMA as nitrogen sources when glucose was present as a carbon and energy source in monocultures but not in purely photoautotrophic cocultures, this suggests that strain KarMa must make a certain energetic investment to utilize these higher methylated forms of MMA that might not be sufficiently provided in the exudates of *P. tricornutum* under photoautotrophic laboratory conditions.

Regarding energy metabolism, it is unknown whether strain KarMa’s growth is affected by the formaldehyde that arises from methylamine degradation. Further, in contrast to what has been found for TMA and TMAO utilization in *R. pomeroyi* (Lidbury et al., 2015), for strain KarMa, we were not able to determine whether methylamines promote growth (Suleiman et al., 2016 and results not shown). In addition, the fact that formate did not increase the growth yield with succinate in strain KarMa (Figure 2) indicates that, in this strain, oxidation of the C1 residue does not significantly contribute to energy conservation. As an additional carbon and energy source supported strain KarMa’s ability to utilize DMA and TMA, this indicates that a phycosphere rich in organic substrates can boost the nitrogen supply from DON. Therefore, when algae release DOC as a substrate for the bacteria dwelling in the phycosphere, this may cause a positive feedback loop for algal growth by stimulating bacterial degradation of DON compounds, such as DMA and TMA. This positive feedback loop might also explain why *P. tricornutum* reached higher abundances in the condition with the methylamine mixture containing 0.66 mM MMA compared to the condition with the equimolar amount of MMA as the sole nitrogen source (Figure 2A). In this respect, glycolate, a frequent side product of algal assimilation from photorespiration (Landa et al., 2017; Schada von Borzyskowski et al., 2019), could easily provide this function as an energy substrate in the phycosphere; in the case of strain KarMa, glycolate could only be used as an energy substrate. In agreement with this, the genome of strain KarMa contains the glycolate oxidase genes for dissimilating glycolate, but it lacks the gene for hydroxyaspartate aldolase, the key enzyme in the β-hydroxyaspartate cycle responsible for assimilating glycolate (Schada von Borzyskowski et al., 2019).

Our results show that ammonium cross-feeding was possible not only with our model organism, strain KarMa, which was isolated as a cross-feeder, but also with several selected *Rhodobacteraceae*-type strains, and it was possible not only from the degradation of methylamines but also from that of GBT. As *Rhodobacteraceae* are known to be algal associated (Buchan et al., 2014), this result indicates that this cross-feeding may be a general phenomenon in the phycosphere that can enhance primary production by N cycling.

This finding was especially apparent for GBT, which served as an ammonium cross-feeding substrate for all *Rhodobacteraceae* strains tested. GBT is a quaternary methylated amine that serves as an osmolyte for diatoms under nitrogen replete conditions and may be excreted in large quantities in the case of rapidly decreasing salinity in order to ensure intracellular osmotic pressure (Kirst, 1990; Keller et al., 1999a, b; Hockin et al., 2012). As GBT can be reused as osmolyte but, apparently, not metabolized by algae (Torstensson et al., 2019), any nitrogen that has been fixed into GBT is not available for other purposes in algae; additionally, GBT might be easily lost by convection. Thus, diatoms may benefit from maintaining in their phycosphere *Rhodobacteraceae* that can metabolize GBT and provide ammonium to the algae, thus creating a short nitrogen cycle in the phycosphere. In this respect, the diffusion-controlled transport of metabolites in the phycosphere that leads to a transient enrichment of GBT and ammonium closing the algal cell is of high importance; a similar mechanism has been modeled for the release and degradation of dimethylsulfide released by unicellular marine algae (Lavoie et al., 2018).

In accordance with the adaptation of the bacterial phycosphere community to GBT metabolism, a previous study found higher levels of GBT-transporter transcripts of *R. pomeroyi* when cocultivated with *T. pseudonana* than in bacterial monocultures (Durham et al., 2017). In our study, although cocultures of strain KarMa and *P. tricornutum* with GBT showed a slower growth rate for the diatom than in cocultures with MMA, high amounts of ammonium were present; so far, it is unknown what hindered the growth of *P. tricornutum*. However, since bacteria do positively influence phytoplankton but may turn to competition or parasitism under changing conditions (e.g., Amin et al., 2012; Mayers et al., 2016), it could be possible that strain KarMa competed with the diatoms for nutrients other than ammonium or exerted any inhibitory effects on diatom growth.

Interestingly, the *Rhodobacteraceae* strains showed differences regarding their ability to use methylamine in monocultures and in cocultures with *P. tricornutum.* Although the high similarity of the gene clusters for methylamine degradation of strain KarMa and *C. halophilus* caused us to predict a similar interaction between these bacteria and *P. tricornutum*, we observed no ammonium cross-feeding for *C. halophilus*, even though MMA and the methylamines were degraded in monocultures. Also for *S. pseudonitzschiae* with MMA and for five of the *Rhodobacteraceae* family members with the methylamine mixture, we did not observe the anticipated cross-feeding of ammonium. As another interesting finding, diatom abundance with the methylamine mixture was much higher for *R. denitrificans* than for strain KarMa cocultures, indicating that ammonium cross-feeding may be subject to regulatory processes. Durham et al. (2017) and Landa et al. (2017) both reported for *R. pomeroyi* that diatom-specific transcription levels of cell communication and signaling genes were present; in *R. pomeroyi* and possibly other *Rhodobacteraceae* family members that interact with diatoms, some of these regulatory genes may also be involved in the cross-feeding process. In addition, under nitrogen-replete conditions, the metabolic interactions may also change, e.g., the amount of DOC released from cells might change. As diatoms are well adapted for nitrate competition (Lomas and Gilbert, 2000; Allen et al., 2006, 2011), their metabolism might be regulated accordingly. Thus, when nitrate was present, it may have changed the overall amount or composition of carbon sources present for strain KarMa, which might be why lower bacterial abundance was observed with nitrate. With respect to the enormous organismic diversity and the fluctuating conditions in the natural habitats of marine microorganisms, we must acknowledge that our model systems can only reflect a small fraction of this *in situ* complexity. Nevertheless, our study highlights that some nitrogen-cycling processes in the phycosphere are based on the bacterial metabolism of methylated amines.

## Data Availability Statement

All datasets generated for this study are included in the article/Supplementary Material.

## Author Contributions

KZ conceptualized the study together with BP, planned and performed most of the experiments, wrote a first draft of the manuscript, and also supervised KH. KH conducted the experiments regarding substrate spectra. BP was involved in conceptualization of the study and planning of the experiments and wrote the final version of the manuscript. All authors contributed to the article and approved its submission.

## Conflict of Interest

The authors declare that this study received funding from Evonik Resource Efficiency GmbH. The funder had the following involvement in the study: decision to submit the study for publication.
