# Supplementary material for: Evidence of Interdomain Ammonium Cross-Feeding From Methylamine- and Glycine Betaine-Degrading Rhodobacteraceae to Diatoms as a Widespread Interaction in the Marine Phycosphere
Source: Front Microbiol. 2020 Oct 6;11:533894. doi: 10.3389/fmicb.2020.533894 (PMC7574528; doi:10.3389/fmicb.2020.533894)
Supplement: Supplementary file 1 [file Data_Sheet_1.pdf]

Supplementary Material to

# Evidence of interkingdom ammonium cross-feeding from methylamine- and glycine betaine-degrading *Rhodobacteraceae* to diatoms as a widespread interaction in marine habitats

**Karsten Zecher, Kristiane Rebecca Hayes, Bodo Philipp**

University of Münster, Institute for Molecular Microbiology and Biotechnology, Corrensstr. 3, D-48149 Münster, Germany

**Supplementary Table 1:** Putative proteins involved in DMA and TMA degradation identified by pBLAST search analysis using *R. pomeroyi* sequences as query (Lidbury *et al.*. 2017).

| Protein | Accession number from *R. pomeroyi* | Accession number strain KarMa | e-value | Identity [%] | Query coverage [%] |
|---|---|---|---|---|---|
| Tmm | Spo1551 | WP_072703413.1 | 0 | 81 | 99% |
| Tdm | Spo1562 | WP_072703415.1 | 0 | 64 | 100% |
| DmmD | Spo1579 | WP_072703363.1 | 0 | 64 | 100% |
| DmmA | Spo1580 | WP_072703360.1 | $4.00e^{-63}$ | 53 | 93% |
| DmmB | Spo1581 | WP_072703357.1 | $3.00e^{-110}$ | 49 | 95% |
| DmmC | Spo1582 | WP_072703354.1 | 0 | 72 | 97% |

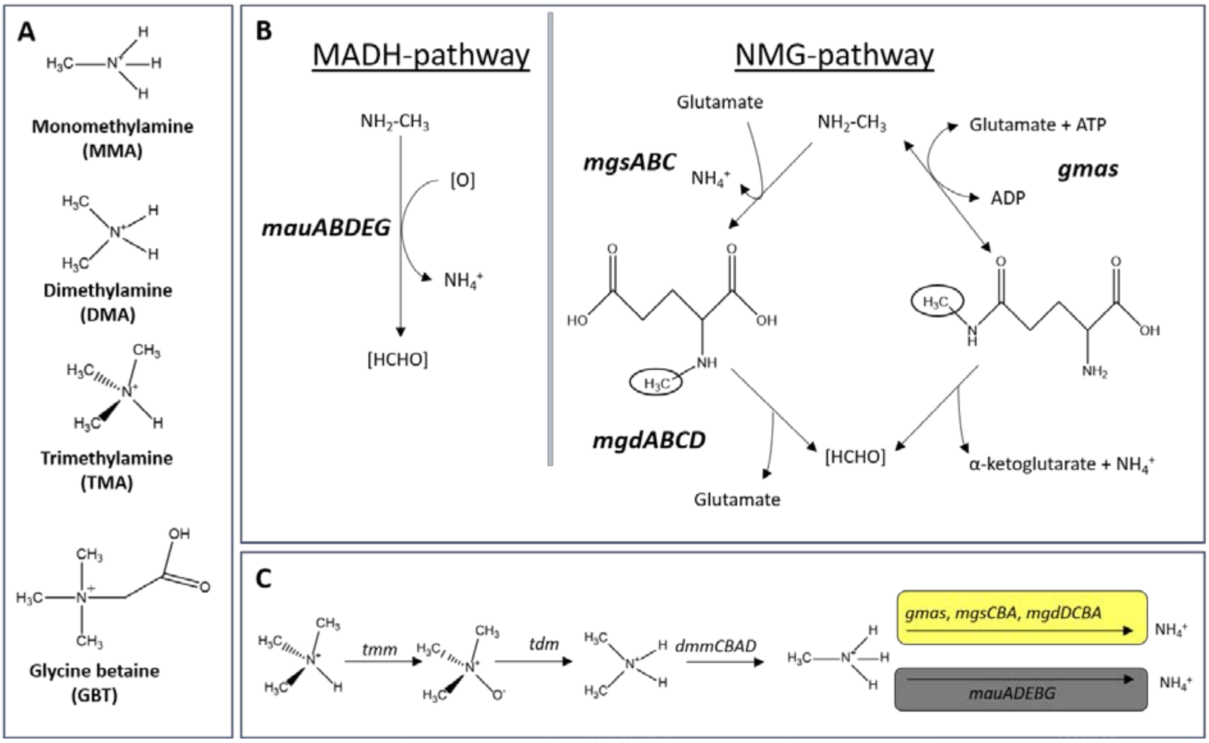


**Supplementary Figure 1:** Bacterial metabolism of methylamines.
(**A**) Chemical structures of methylamines and of glycine betaine; (**B**) the methylamine dehydrogenase (MADH) pathway and the N-methylglutamate (NMG) pathway for MMA degradation (adapted from Latypova et al, 2010; https://doi.org/10.1111/j.13652958.2009.06989.x); (**C**) degradation pathway for TMA and DMA to MMA (adapted from Lidbury et al., 2017; https://doi.org/10.1038/ismej.2017.31)

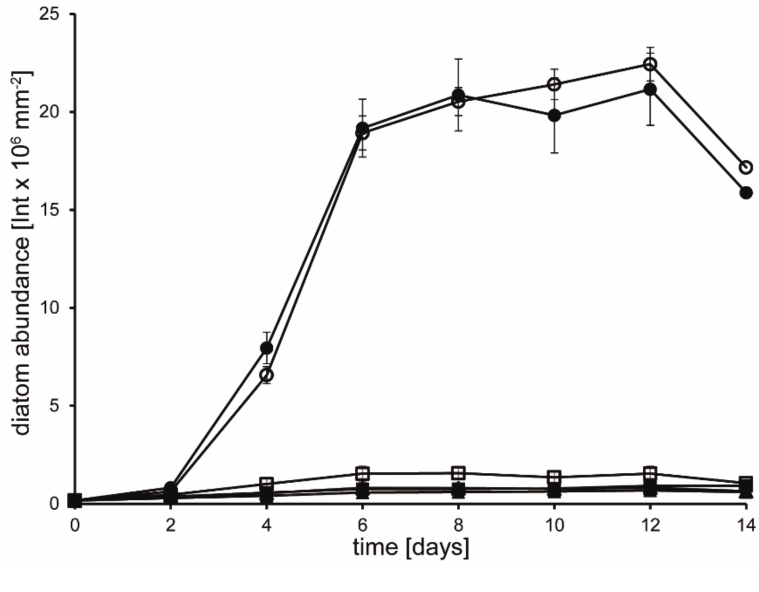


**Supplementary Figure 2:** Diatom abundance of *P. tricornutum* in single (open symbols) and co-cultures with strain KarMa (closed symbols) with putrescine (▲), spermidine (■) and urea (●) as sole nitrogen source in 24-well plates. Error bars indicate standard deviation (n=3).

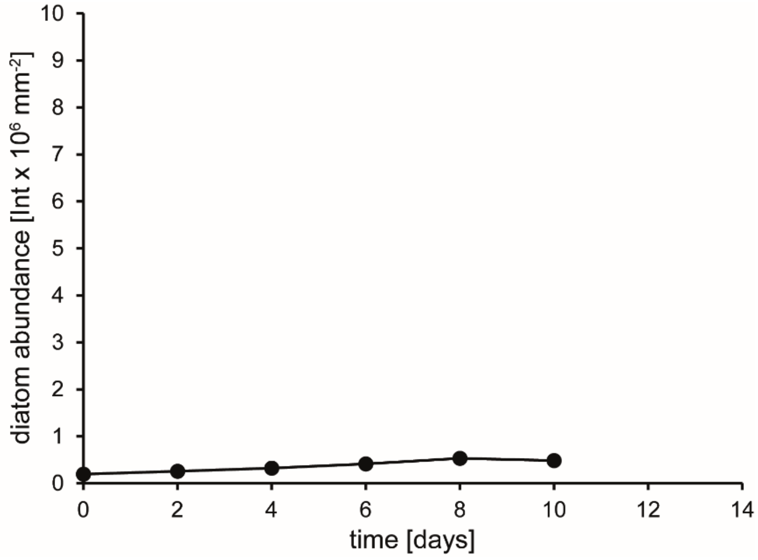


**Supplementary Figure 3.** Growth of *P. tricornutum* in single culture with GBT as sole nitrogen source. Error bars indicate standard deviation (n=3).

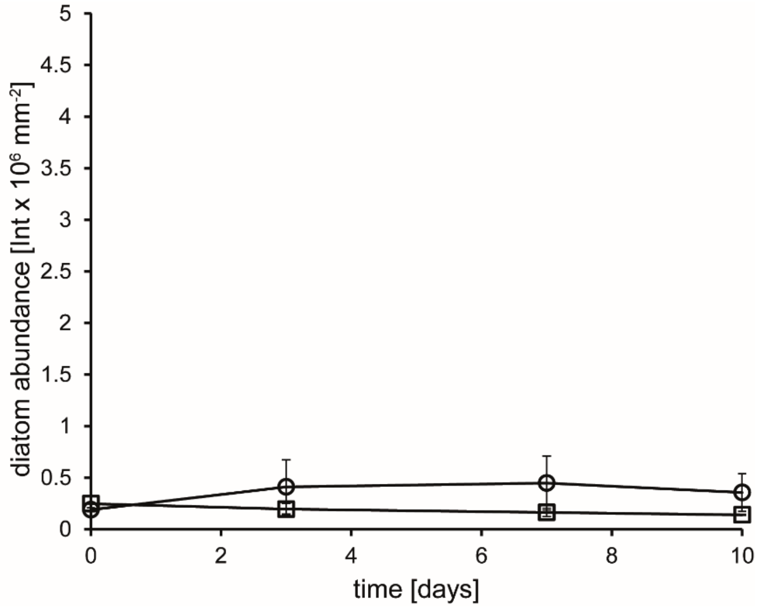


**Supplementary Figure 4:** Growth of the diatoms *A. coffeaeformis* and *T. pseudonana* with 2 mM MMA in mono-cultures. Error bars indicate standard deviation (n=3).
